# Hepatitis a virus genotypes and strains from an endemic area of Europe, Bulgaria 2012–2014

**DOI:** 10.1186/s12879-017-2596-1

**Published:** 2017-07-14

**Authors:** Roberto Bruni, Stefania Taffon, Michele Equestre, Eleonora Cella, Alessandra Lo Presti, Angela Costantino, Paola Chionne, Elisabetta Madonna, Elitsa Golkocheva-Markova, Diljana Bankova, Massimo Ciccozzi, Pavel Teoharov, Anna Rita Ciccaglione

**Affiliations:** 1 National Reference Laboratory for HAV, Viral Hepatitis Unit, Department of Infectious Diseases, Istituto Superiore di Sanità, Rome, Italy; 2 Department of Neurosciences, Istituto Superiore di Sanità, Rome, Italy; 3 Epidemiology Unit, Department of Infectious Diseases, Istituto Superiore di Sanità, Rome, Italy; 4 Department of Virology, National Center of Infectious and Parasitic Diseases, Sofia, Bulgaria

**Keywords:** Hepatitis a virus, HAV, Hepatitis, Phylogenetic analysis, Sequencing, Bulgaria

## Abstract

**Background:**

Hepatitis A virus (HAV) infection is endemic in Eastern European and Balkan region countries. In 2012, Bulgaria showed the highest rate (67.13 cases per 100,000) in Europe. Nevertheless, HAV genotypes and strains circulating in this country have never been described. The present study reports the molecular characterization of HAV from 105 patients from Bulgaria.

**Methods:**

Anti-HAV IgM positive serum samples collected in 2012–2014 from different towns and villages in Bulgaria were analysed by nested RT-PCR, sequencing of the VP1/2A region and phylogenetic analysis; the results were analysed together with patient and geographical data.

**Results:**

Phylogenetic analysis revealed two main sequence groups corresponding to the IA (78/105, 74%) and IB (27/105, 26%) sub-genotypes.

In the IA group, a major and a minor cluster were observed (62 and 16 sequences, respectively). Most sequences from the major cluster (44/62, 71%) belonged to either of two strains, termed "strain 1" and "strain 2", differing only for a single specific nucleotide; the remaining sequences (18/62, 29%) showed few (1 to 4) nucleotide variations respect to strain 1 and 2. Strain 2 is identical to the strain previously responsible for an outbreak in the Czech Republic in 2008 and a large multi-country European outbreak caused by contaminated mixed frozen berries in 2013.

Most sequences of the IA minor cluster and the IB group were detected in large/medium centers (LMCs). Overall, sequences from the IA major cluster were more frequent in small centers (SCs), but strain 1 and strain 2 showed an opposite relative frequency in SCs and LMCs (strain 1 more frequent in SCs, strain 2 in LMCs).

**Conclusions:**

Genotype IA predominated in Bulgaria in 2012–2014 and phylogenetic analysis identified a major cluster of highly related or identical IA sequences, representing 59% of the analysed cases; these isolates were mostly detected in SCs, in which HAV shows higher endemicity than in LMCs. The distribution of viral sequences suggests the existence of some differences between the transmission routes in SCs and LMCs.

Molecular characterization of an increased number of isolates from Bulgaria, regularly collected over time, will be useful to explore specific transmission routes and plan appropriate preventing measures.

## Background

Hepatitis A virus (HAV) is one of the major causes of acute hepatitis throughout the world and causes substantial morbidity in both developed and developing countries [1, 2]. On the basis of genome sequence divergence, all viruses infecting humans have been classified in three genotypes (I, II, III), further divided into two sub-genotypes (A and B). Genotypes and subtypes tend to show different geographic distribution [3].

HAV is mainly transmitted by the faecal-oral route. HAV can survive for long in water and numerous waterborne epidemics have been observed following consumption of contaminated drinking water [4], food produce [5, 6], or shellfish [7].

The infection is usually asymptomatic or mild in children under five years, while in adults more frequently occurs with symptoms and jaundice. Clearance of infection confers lifelong immunity. In high endemicity countries, HAV infection is acquired in early childhood and most adult population is positive for anti-HAV IgG and protected from re-infection. In contrast, in low endemicity countries most adult population is susceptible: as a result, infections are more likely to occur in adults, in which are frequently symptomatic [8].

In Europe, the epidemiology of the HAV infection is characterized by countries with variable endemicity. In 2012, the European Centre for Disease Prevention and Control (ECDC) reported a mean rate of infection in Europe of 2.6 cases per 100,000 inhabitants (13,156 total confirmed cases). The majority of them were from an endemic area which included Eastern European and Balkan region countries [9]. Among them, Bulgaria is a country with intermediate hepatitis A endemicity, with annual incidence between 25 and 94 cases per 100,000 population (data since 2000). In 2011 and 2012 the cases increased to 75.88/100,000 (5588 cases) and 67.13/100,000 (4919 cases), respectively, then decreased to 25.05/100,000 in 2013 (1825 cases) [10].

In 2011–2013, the most affected population group was children aged 5–9 years, followed by the 10–14 years and 1–4 years age groups, which are the main risk groups for the disease in Bulgaria in general. During this time, 28 outbreaks of hepatitis A were detected in different Bulgarian districts, affecting dominantly minority Roma neighbourhoods [9, 10]. The prevalence of anti-HAV antibodies in general population in Bulgaria is poorly known. The only available data were from a small study carried out in Plovdiv, the second largest town in Bulgaria, in which two quarters with very different hygienic and sanitary conditions were sampled [11]. The mean seroprevalence was 68.3%, but it reached 90.2% in the quarter with poor hygienic and sanitary conditions and 44.8% in the other with normal conditions [11]. Vaccination in Bulgaria is recommended for people at risk [12]; however, targeted immunization campaigns are launched in response to outbreaks, such as in 2006 [13].

Molecular analysis of HAV isolates has proved to be crucial for understanding the demographic, social and environmental factors that have given rise to the current epidemiological patterns in Europe. Several studies described circulation of different HAV genotypes from both autochthonous and travel-related cases [14–21]. Nevertheless, HAV strains from Bulgaria and other European endemic countries have never been described. This gap delays the comprehension of the local transmission routes and the implementation of control measures in areas that urgently need them.

In the present study, serum samples from 105 patients with acute hepatitis A, collected in different towns and villages in Bulgaria during 2012–2014, were analysed by nested RT-PCR and sequencing to characterize HAV isolates. Phylogenetic analysis of sequences was performed to describe, for the first time, HAV genotypes and strains circulating in Bulgaria.

## Methods

### Patients

The Department of Virology of the National Center of Infectious and Parasitic Diseases in Bulgaria receives serum samples from patients with acute hepatitis (according to the clinical definition by World Health Organization (WHO) [22]) from all over the country for laboratory testing. One hundred and five anti-HAV IgM positive sera from cases occurred during 2012–2014 in several towns/villages, mostly in West Bulgaria, had been randomly stored and were available for the present study. Anti-HAV IgM antibodies had been detected by the HAV IgM ELISA kit (Dia.Pro, Diagnostic BioProbes s.r.l., Italy). All the 105 cases were classified as confirmed HAV infections on the basis of specific IgM antibody detection according to the recommended case definition by WHO [22]. Among them, 91.4% were Bulgarian, 51.4% were males, the mean age of the patients was 21 years, ranging from 2 to 78. HAV characterization by nested RT-PCR and sequencing was carried out in Italy at the National Reference Laboratory for Hepatitis Viruses - Istituto Superiore di Sanità (NRL-ISS) (see next paragraph). Informed consent for participation in the study had been obtained before patient discharge. The study was approved by the Ethics Committee at the National Center of Infectious and Parasitic Diseases, Sofia, Bulgaria.

### Nested RT-PCR and sequencing

Viral RNA was extracted from 140 μL serum by using the QIAmp viral RNA extraction kit (Qiagen, Hilden, Germany). One sixth extracted RNA (10 μL) was reverse transcribed by the SuperScript III First-Strand Synthesis System for RT-PCR (Invitrogen) with random hexamers. Nested PCR was carried out as previously reported [14, 15]. To control for possible PCR contamination, negative control sera were included in each run: they were extracted, reverse transcribed and amplified by nested PCR along with the sera under study. Double strand sequencing of purified PCR products obtained from human sera was carried out by using the GenomeLab Dye Terminator Cycle Sequencing (DTCS) Quick Start Kit and an automated DNA sequencer (both kit and instrument by Beckman Coulter, Inc., Fullerton, CA). The sequencing reaction was carried out according to the manufacturer instructions. In brief, 20–40 ng purified DNA was used as template with 3.2 pmoles of primer and DTCS Quick Start Master Mix, in a 20 μL final volume. The reaction was subjected to 30 cycles of: 96 °C, 20 s; 50 °C, 20 s; 60 °C, 4 min.

The sequenced region encompassed the VP1/2A region of HAV genome (460 nt, positions 2915 to 3374 in the HM-175 reference sequence Acc.No. NC_001489).

### Sequence analysis

Genotype was assigned by phylogenetic analysis of a dataset containing 105 HAV sequences from Bulgaria [GenBank:KX858970 to KX859074] plus 13 HAV reference sequences representative of genotypes and sub-genotypes (sub-genotype IA [GenBank:EF406357; KC182590; X75215; X83302], sub-genotype IB [GenBank:AF314208; M20273; NC001489; AF268396], sub-genotype IIA [GenBank:AY644676], sub-genotype IIB [GenBank:AY644670], sub-genotype IIIA [GenBank:AJ299464; KF706410], sub-genotype IIIB [GenBank:AB258387]).

Phylogenetic relationships were analysed by constructing a Neighbor-Joining phylogenetic tree by MEGA 6.0 [23]. The best substitution model (T92 + G) was selected by analysis of sequences with the Models tool in MEGA. Tree reliability was assessed by setting bootstrap replicates to 1000. Bootstrap values >70 were considered significant.

Mean intra-group identity level was calculated from the output identity matrix obtained by the Sequence Identity Matrix function in BioEdit 7.2.5 [24].

## Results

### Distribution of patients according to age and size of urban centers

According to official data of 2011 census, urban centers were classified in large to medium centers (LMCs) (>30,000 inhabitants, range 44,000–1,203,000) and small centers (SCs) (<30,000 inhabitants, range 132–27,000). One hundred and five patients with acute HAV infection were evaluated in the study. Among them, 46 (43.8%) were from large to medium centers (LMCs) and 50 47.6%) from small centers (SCs). Nine subjects (8.6%), of unknown nationality, were from a refugee camp in Bulgaria. The age was known for 102 patients and their classification into three age categories (Children: 0–12; Teens: >12 to 19; Adults: >19) indicated that 48 (47.1%) were children, 13 (12.7%) teens and 41 (40.2%) adults.

**Fig. 1:**
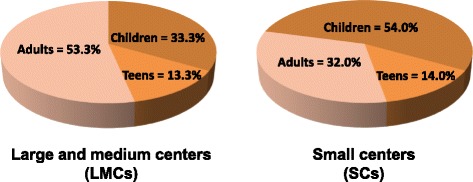
Distribution of molecularly characterized hepatitis A cases according to age categories and size of urban centers. The distribution in three age groups (Children: 0–12 years; Teens: >12 to 19 years; Adults: > 19 years) according to the size of urban centers (Large and medium centers: >30,000 inhabitants, range 44,000–1,203,000; Small centers: <30,000 inhabitants, range 132–27,000) is reported

Distribution of hepatitis A cases according to the size of urban centers revealed an opposite age distribution pattern in LMCs and SCs (Fig. 1). The percentages of children and adults were 33.3% and 53.3%, respectively, in patients from LMCs, while they were 54.0% and 32.0% in patients from SCs (*p* < 0.05, Fisher’s exact test). The percentage of teens was similar in the two groups.

### Genotyping of HAV strains

**Fig. 2:**
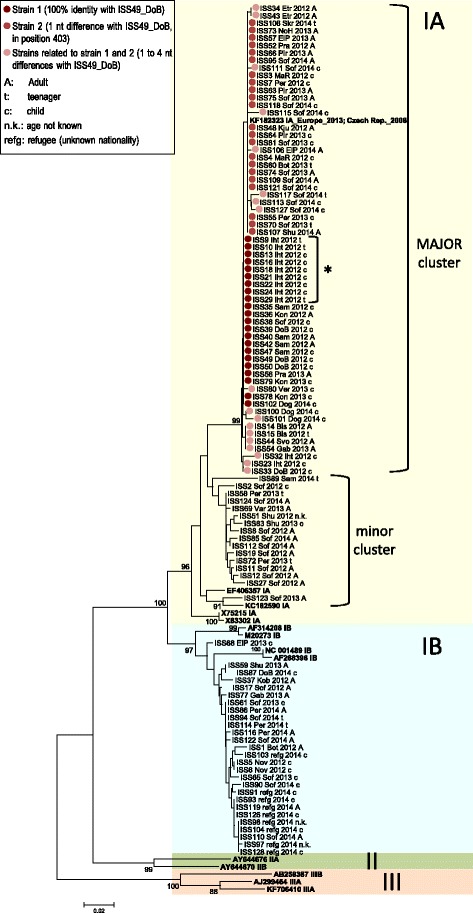
Neighbour-Joining phylogenetic tree (Substitution model: T92 + G) of the 105 HAV isolates from Bulgaria. For each sequence, the town/village (Bis: Bistritsa; Bot: Botevgrad; DoB: Dolna Banja; Dog: Doganovo; ElP: Elin Pelin; Etr: Etropole; Gab: Gabrovo; Iht: Ihtiman; Kju: Kjustendil; Kob: Kostinbrod; Kon: Kostenets; MaR: Manaselska Reka; NoH: Novi Han; Nov: Novachene; Per: Pernik; Pir: Pirdop; Pra: Pravetz; Sam: Samokov; Shu: Shumen; Skr: Skravena; Sof: Sofia; Svo: Svoge; Var: Varna; Ver: Verinsko; refg: refugee, village information not available), the isolation year (2012, 2013, 2014) and the age group of the patient (A: Adult; t: teenager; c: child; n.k.: not known) are reported after the sequence ID. Reference strains with known genotype (IA, IB, IIA, IIB, IIIA, IIIB) were included in the analysis and are reported (in bold) by Accession number followed by the sub-genotype they belong to. Significant bootstrap values are reported. The asterisk shows a small outbreak of 9 cases from Ihtiman occurred between 25 June and 8 August 2012 and involving 6 children and 3 teenagers: the complete nucleotide identity of the isolates confirms a local small outbreak caused by the same strain

Phylogenetic analysis of the 105 HAV isolates from Bulgaria and reference sequences with known genotype revealed two main groups corresponding to the IA (*n* = 78) and IB (*n* = 27) sub-genotypes (Fig. 2). No genotype II or III isolates were observed.

In the IA group two main statistically supported clusters can be observed (Fig. 2, labelled as “MAJOR cluster” and “minor cluster”). The major cluster included sequences (*n* = 62, 79.5% of IA isolates) showing a very high intra-cluster identity level (mean value 99.6%¸ range 98%–100%).

The minor cluster contained IA sequences (*n* = 16, 20.5% of IA isolates) characterized by a lower intra-cluster identity level (mean value 98.1%¸ range 93.7%–100%).

In the IB group, the 27 sequences showed a high identity level (mean value 99.1%¸ range 98%–100%). Among the IB isolates from 9 patients living in a refugee camp in Bulgaria, 2 small clusters (each containing 3 isolates) of identical sequences were observed. Although the sequences from refugees showed a tendency to cluster separately from the other IB isolates, bootstrap values were not significant: sequences from refugees showed high identity (1 to 4 nt differences) with several sequences from Bulgarian patients circulating in the same (2014) and in the previous years (2012 and 2013) suggesting that refugees got infected with local strains.

To include in the phylogenetic analysis also the proposed sub-genotype IC [17], a separate analysis limited to a shorter region (261 nt) had to be carried out; in fact the available reference IC sequences only partially overlapped with the region sequenced in the present study. IC isolates formed a separate cluster, intermediate between IA and IB; however, none of the Bulgarian isolates grouped with them (data not shown).

### Nucleotide variations in strains from the IA major cluster

To better compare HAV isolates from the IA major cluster, one of them, the ISS49_DoB, was chosen as reference sequence. Based on this, the 62 isolates from the major cluster were classified into three groups as follows (see also Fig. 2): (1) “strain 1” group, including 22 (35.5%) sequences (21 sequences showing 100% identity with ISS49_DoB plus the reference sequence itself); (2) “strain 2” group, including 22 (35.5%) sequences presenting only 1 nucleotide (nt) difference with ISS49_DoB, with all isolates showing the same nt difference in the same position (nt 403, C *→* T, numeration of [GenBank:KF182323]); strain 2 is identical to a strain (reference sequence [GenBank:KF182323] in Fig. 2) previously reported to be responsible for both an outbreak in the Czech Republic in 2008 and a large European outbreak caused by mixed frozen berries in 2013 [15]; (3) “strains related to strain 1 and 2” group, including 18 (29%) sequences showing 1 to 4 nucleotide differences versus ISS49_DoB (individual nucleotide variations were scattered in different positions) (Fig. 2, see the box). Overall, strain 1 and strain 2 represent 44/62 (71%) isolates of the major cluster; 12 distinct variants could be recognised in the remaining 18/62 (29%) isolates of this cluster.

By linking epidemiological and sequence data, a small outbreak of 9 cases could be clearly identified in the major cluster. All cases were from Ihtiman village and occurred between 25 June and 8 August 2012, involving 6 children and 3 teenagers: the complete identity of the isolates confirms a local small outbreak caused by the strain 1 during a period of two months (Fig. 2, MAJOR cluster, labelled by the asterisk).

### Distribution of HAV genotypes and strains in urban centers of different size

**Fig. 3:**
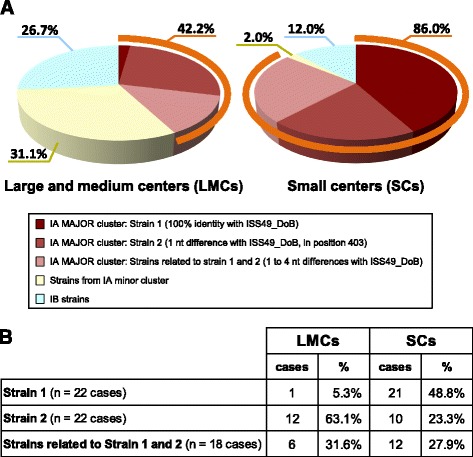
Distribution of strains according to the size of urban centers. **a** Distribution of the strains from the IA Major cluster, IA minor cluster and IB cluster according to the size of urban centers (see the legend of Fig. 1). The percent values are shown. **b** Detail of the number of cases and relative frequency of the strains from the IA Major cluster (strain 1, strain 2 and strains related to strain 1 and 2) in LMCs and SCs

Distribution of HAV genotypes according to the size of the town/village of patients showed that the percentages of isolates belonging to IA major cluster, IA minor cluster and IB group were 42.2%*,* 31.1% and 26.7%, respectively, in patients from LMCs and 86.0%*,* 2.0% and 12.0% in patients from SCs (Fig. 3a). Most isolates from the IA major cluster (43/62, 69.4%) were identified in patients from SCs. In contrast, all but one strains from the IA minor cluster were observed in patients from LMCs, suggesting that different transmission routes, linked to urbanization, operate in these cases. Among the IB sub-genotype, isolates were identified in both LMCs and SCs, however the frequency was higher in patients from LMCs (26.7% in LMCs vs 12% in SCs) (Fig. 3a). Analysis of the 62 isolates from the IA major cluster revealed a different frequency of strain 1 and strain 2 in LMCs and SCs (Fig. 3b): strain 1 largely prevailed in SCs (5.3% in LMCs vs. 48.8% in SCs), strain 2 prevailed in LMCs (63.1% in LMCs vs. 23.3% in SCs) (*p* < 0.001, Fisher’s exact test); in contrast, the strains related to strain 1 and 2 showed similar frequency (31.6% vs. 27.9%).

**Fig. 4:**
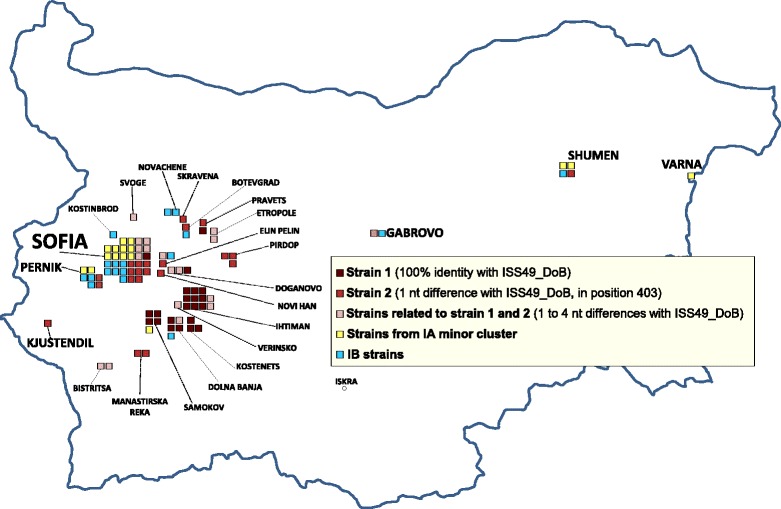
Geographical localization of the characterized strains in Bulgarian towns/villages. The name of centers >30,000 inhabitants is reported in larger font size than the name of centers <30,000 inhabitants

Analysis of the geographical localization of the strains in Bulgaria map showed that strain 1 was preferentially detected in a restricted area south-east of Sofia, strain 2 and strains related to strain 1 and 2 showed a more scattered distribution, whereas most strains from the IA minor cluster and IB strains were observed in large towns such as Sofia, Pernik, Shumen, Gabrovo and Varna (Fig. 4).

## Discussion

The ECDC surveillance report of food- and waterborne diseases, published in 2014, defined Bulgaria as the European country that reported the highest number of hepatitis A cases in 2011 (5587 cases) and 2012 (4919 cases). Investigation of a large outbreak in Bulgaria, occurred in 2012 in the Iskra village in the Plovdiv region, indicated that unregulated and/or frequently failing water supply and sewage system are associated with virus spread in this area [9]. Despite the level of endemicity, molecular epidemiology of HAV in Bulgaria has never been described. Thus, the studied samples represent an extremely rare resource, if not unique, to obtain information on HAV strains that have circulated and might still be circulating in an Eastern Europe country. The results of the present study represent the first analysis of the HAV genotypes and strains circulating in Bulgaria.

Analysis of the age distribution showed that patients were younger in SCs than in LMCs, suggesting different endemicity among these areas. In SCs, high HAV endemicity results in a large proportion of adults protected by antibodies developed in childhood and, thus, HAV circulation mainly in children. High endemicity in SCs is likely related to poor hygienic conditions and health education of the population, as suggested by a study investigating an outbreak occurred in 2012 in a SC (Iskra village) [25].

Molecular characterization of HAV from 105 acute hepatitis A cases, collected between 2012 and 2014, indicated that both IA and IB sub-genotypes circulate in Bulgaria; however, about 74% of the isolates were assigned to sub-genotype IA that, thus, seems to be dominant.

Interestingly, IA strains grouped in two distinct phylogenetic clusters. The major cluster, that included 79.5% of the IA cases and 59% of the total cases, contained highly related viruses. Sequence analysis indicated that isolates were either identical (at least over the analysed genomic region), as observed in strain 1 or strain 2 groups, or showed very few (1 to 4) nucleotide variations. Strain 1 and strain 2 groups differed each other for a single specific nucleotide, that was T in strain 1 and C in strain 2. The very low level of variability observed in the IA major cluster might be the result of a common origin of the two main strains and of their spread in the local population, with random generation of new variants, highly related to the main strains, in the course of endemic and epidemic circulation.

The distribution of strains according to urban size (Fig. 3b) and the geographical localization of the involved urban centers (Fig. 4) indicate that strain 1 preferentially circulated in small urban centers and villages south-east of Sofia, where transmission was likely maintained by poor water supply and lack of infrastructures. Although the relative frequency of strain 2 was higher in LMCs than in SCs (Fig. 3b), strain 2 showed wider geographical distribution than strain 1 (Fig. 4) and it was detected both in large urban centers, such as Sofia, Pernik and Shumen, and in small villages.

A completely different pattern of circulation was observed for isolates from the IA minor cluster and from the IB cluster. In fact, all but one patients infected with strains from the IA minor cluster and two thirds of cases infected with IB strains were from LMCs (Fig. 3a), suggesting that different transmission routes, linked to urbanization, might operate in these cases (e.g. consumption of shellfish or other food, contaminated in the same or other countries).

Assuming the distribution of HAV genotypes and strains reported in the present study roughly represents isolates circulating in Bulgaria in 2012–2014, it is conceivable that the IA strains from the IA major cluster, accounting for 59% of sequenced cases, were responsible for the epidemic peak observed in Bulgaria in 2011 and 2012 and, most likely, for the multiple outbreaks detected in different Bulgarian districts during those years [9, 10].

## Conclusions

This is the first study describing the HAV genotypes and strains circulating in Bulgaria, one of the European countries with intermediate endemicity. Genotype IA predominates, with several highly related strains. Further studies need to be carried out to increase the number of molecularly characterised isolates from Bulgaria and from other countries of Eastern Europe that, by a phylogenetic approach, can be useful to investigate the factors underlying the spread of HAV infections and to implement appropriate preventing measures, at both national and European level.

## Abbreviations

HAV: Hepatitis A Virus
LMCs: Large/Medium Centers
RT-PCR: Reverse Transcription and Polymerase Chain Reaction
SCs: Small Centers
